# Isolation of Pasteurella pneumotropica in a Hand Phlegmon Following a Human Bite

**DOI:** 10.7759/cureus.51408

**Published:** 2023-12-31

**Authors:** Yassine Ben Lahlou, Abdelouahab Erraji, Elmostapha Benaissa, Mariama Chadli, Mostapha Elouennass

**Affiliations:** 1 Bacteriology, Mohammed V Military Training Hospital, Rabat, MAR

**Keywords:** pasteurellosis, bite, human bite, phlegmon, pasteurella pneumomotropica

## Abstract

*Pasteurella* is a commensal microorganism found in the mucous membranes of the upper respiratory and digestive tracts of mammals and birds and it rarely affects humans. Human pasteurellosis typically results from infection through bites or scratches from animals, with dogs and cats being the most common sources. However, various vertebrates, such as rats, rabbits, tigers, and lions, can also transmit the infection.

We report a case involving a young woman who developed a hand phlegmon on her right forearm following a rare and unusual human bite during a brawl. Her condition improved after both surgical and medical treatment.

## Introduction

Pasteurellosis is primarily an animal infection that can become apparent in humans when transmitted through bites or scratches [1-3]. It is uncommon to find this infection in the human oral cavity, and even rarer in cases involving a 'biting mouth' [4].

## Case presentation

**Figure 1:**
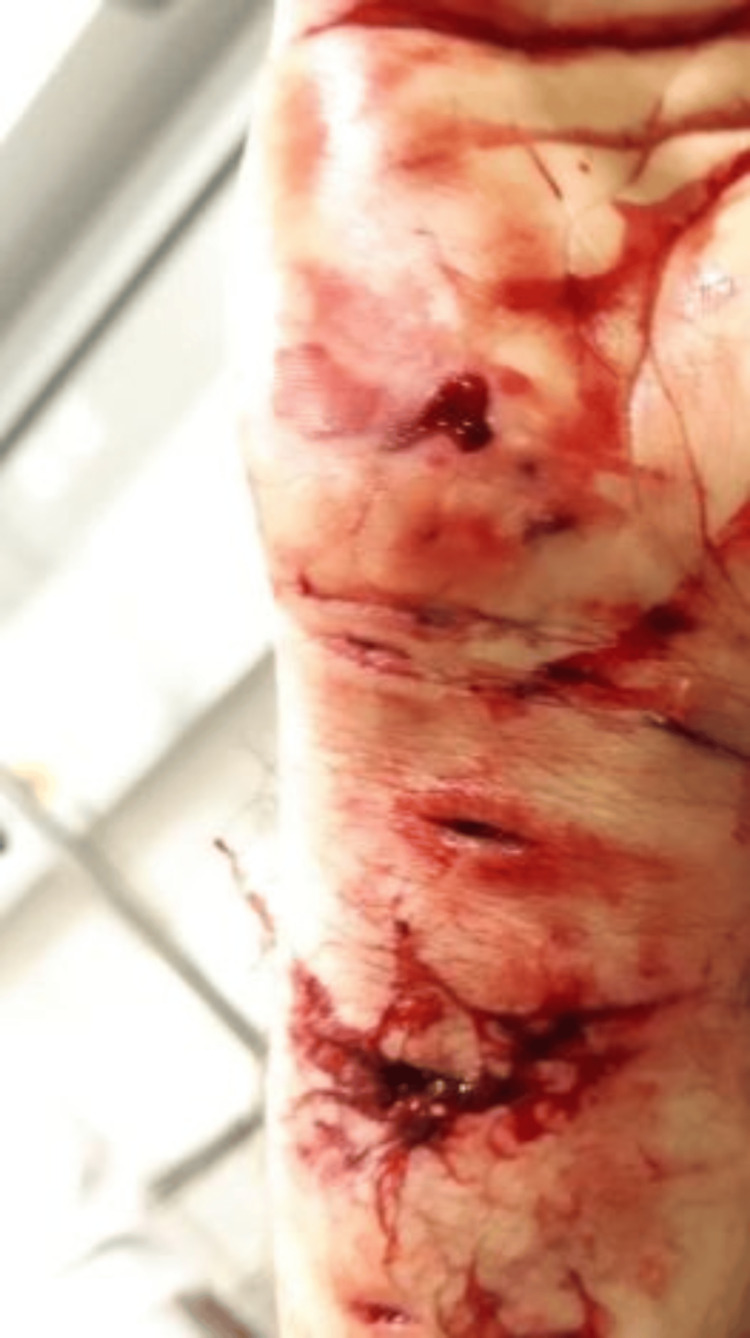
Clinical aspect of the hand following a human bite.

A 29-year-old female patient with no previous significant surgical or family history was admitted to the emergency department of the Mohammed V Military Teaching Hospital in Rabat following a human bite in the right forearm. The clinical examination revealed traumatic dermabrasion lesions and deep lesions (Figure 1). Standard radiography did not reveal vascular, bone, or tendon damage to the forearm. The complete blood count revealed a cell count of 7.8 × 10^9^/L (68% neutrophils, 24% polymorphonuclear lymphocytes) and CRP at 93 mg/L.

A debridement was performed in the operating theater with a bacteriological sampling of the affected area. On the first day of admission, our patient received amoxicillin-clavulanic acid (1g/ 3 IV), and Ciprofloxacin (500 mg 2/day).

**Figure 2:**
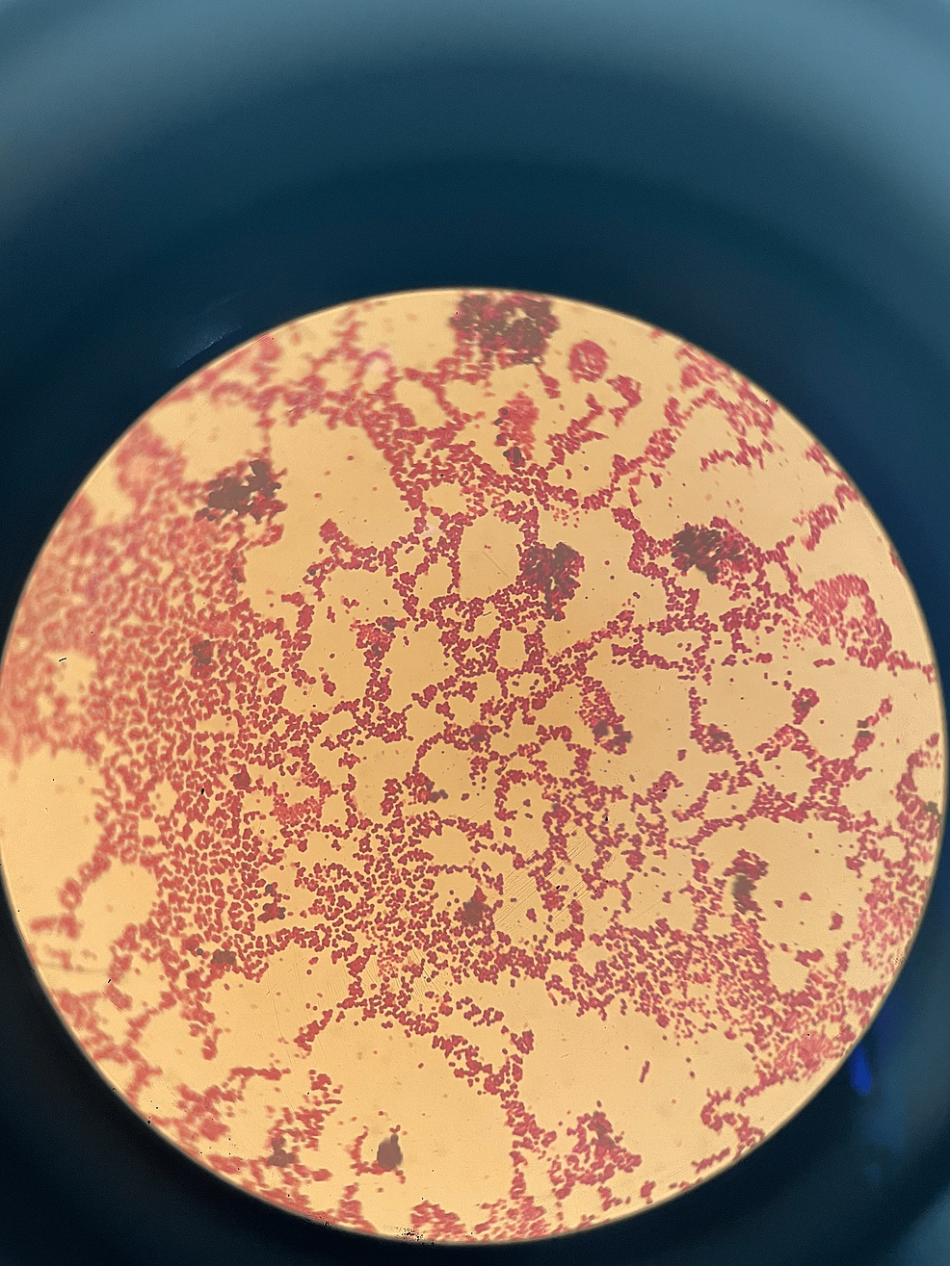
Gram stain of Pasteurella pneumomotropica.

Gram staining revealed the predominance of neutrophilic polymorphonuclear cells, along with the presence of some gram-negative coccobacilli and rare gram-positive cocci. Culture on blood agar and chocolate agar, incubated at 37 °C in an enriched atmosphere of 5-10% CO_2_, led to the isolation of small, even-edged, non-hemolytic colonies after 24 hours of incubation (Figure 2). An Enterobacteriaceae identification gallery API 20E confirmed the species as *Pasteurella pneumotropica* with a 98% match. An antibiogram was carried out by diffusion method in Mueller-Hinton agar medium containing 5% horse blood in compliance with the recommendations of EUCAST 2021. The clinical and biological progress was favorable following antibiotic treatment.

## Discussion

*Pasteurella pneumotropica* was initially described and named by Jawetz in 1948, and then described and studied by Jawetz and Baker in 1950 in lungs of mice, which led to its nomenclature [5]. Morphologically, these are ovoid coccobacilli or short sticks 0.3 to 0.4 mm in diameter and 1 to 2 mm long. Bacterial bodies appear isolated, in pairs, and sometimes form short chains. Gram staining is negative and bipolar staining is common. These bacteria are immobile and they do not form endospores [1,6]. 

Regarding pathogenicity, traditionally, we distinguish pasteurellosis by inoculation into the most frequent, pleuropulmonary pasteurellosis, and other systemic pasteurellosis. In the inoculation pasteurellosis, the acute form develops mainly at the upper limb [7], as in the case of our patient who developed a phlegmon at the forearm. The incubation period is notably short (typically 3 to 6 hours) and never exceeding 24 hours [8,9]. 

The brevity of incubation, which is usually only a few hours, is characteristic [8]. Symptoms are primarily characterized by pain, with the wound becoming edematous, red, with a sap discharge, and rarely purulent. These symptoms are often accompanied by lymphangitis and satellite lymphadenopathy. In the absence of treatment, subacute forms can lead to arthritis and phlegmons of sheaths [1,9]. Respiratory infections occur in cases of immunosuppression, ethylic, smoking, bronchial dilation, and cancer. Sepsis can complicate local infection following a bite or scratch [1,10]. Other, less common manifestations of pasteurellosis include purulent meningitis, most often related to traumatic meningeal or neurosurgical breach [11]. 

The major pathogenicity factor is supported by a peptide toxin encoded by the *tox A* gene, a toxin that has a dermonecrotic and osteolytic power [8].

Compared to the culture of the bacterium, *Pasteurella *can grow on usual media, however, their growth can be facilitated by seeding on better enriched media. In our case, the sample was inoculated onto cooked blood agar, and blood agar incubated at 37°C for 48 hours in a CO_2_ atmosphere. A selective medium containing 2mg/l amikacin and 4mg/l vancomycin was also used [8]. Cultivation of our sample revealed smooth grey colonies of 1-2 mm in diameter. On blood agar, hemolysis was not observed. Species identification was achieved using an API 20E gallery, with a match of 98%.

In the case of a bite, polymorphism is common [12,13], as was observed in our sample. This coexistence of other bacteria with *Pasteurella pneumotropica *raises the problem of its responsibility in the process of suppuration of the wound. In our observation, despite the association with the *Streptococcus* species, a bacterium of the commensal flora of the mouth, *Pasteurella pneumotropica *seems to be considered responsible for the suppuration, because of its predominance in the culture.

Pasteurella is usually sensitive to penicillin G and ampicillin, but β lactamases have been reported in these species which are inactivated by clavulanic acid. Cephalosporins, fluoroquinolones, and cotrimoxazole are active [8, 14]. The first-line treatment in the acute form is amoxicillin 50mg/kg/day. In case of allergy to beta-ctamines, doxycycline is prescribed at the dose of 200 mg per day. 

In our case, *Pasteurella pneumotropica* was sensitive to penicillin which led us to study the minimum inhibitory concentrations of amoxicillin-clavulanic acid and ciprofloxacin for better management.

In addition to surgical treatment, our patient received intravenous amoxicillin-clavulanic acid and ciprofloxacin, resulting in a positive clinical and biological response. 

## Conclusions

A wound resulting from an animal or human bite should raise suspicion for pasteurellosis, and the risk of co-infection by other bacteria should also be considered. In such cases, bacteriological sampling is essential to isolate the strain and establish a sensitivity profile.
